# Hyperadrenergic postural tachycardia syndrome associated with augmented neurovascular transduction

**DOI:** 10.1007/s10286-025-01183-z

**Published:** 2026-01-14

**Authors:** Surat Kulapatana, Luis E. Okamoto, Stefano Rigo, Vasile Urechie, Thomas W. Cayton, Ruijing E. Han, Giris Jacob, William D. Dupont, Raffaello Furlan, Italo Biaggioni, André Diedrich

**Affiliations:** 1 Autonomic Dysfunction Center, Department of Medicine, Division of Genetic Medicine and Clinical Pharmacology, Vanderbilt University Medical Center, 1161 21St Avenue South, Suite S3116 MCN, Nashville, TN 37232-2600 USA; 2 Department of Biomedical Engineering, Vanderbilt University, Nashville, TN USA; 3 Siriraj Vascular Research Center, Department of Physiology, Faculty of Medicine Siriraj Hospital, Mahidol University, Bangkok, 10700 Thailand; 4 Humanitas Clinical and Research Center–IRCCS, Via Alessandro Manzoni, 56, Rozzano, Italy; 5 Department of Biomedical Sciences, Humanitas University, Pieve Emanuele, Italy; 6 Clinical Research Unit, myDoctorAngel Sagl, Bioggio, Switzerland; 7 Department of Internal Medicine F. and J. Recanati Autonomic Dysfunction Center, Tel Aviv Sourasky Medical Center, Faculty of Medicine, Tel Aviv University, Tel Aviv, Israel; 8 Department of Biostatistics, Vanderbilt University Medical Center, Nashville, TN 37232‑2600 USA

**Keywords:** POTS, MSNA, Valsalva, Sympathetic, Hyperadrenergic

## Abstract

**Purpose:**

Muscle sympathetic nerve activity (MSNA) is valuable for managing postural tachycardia syndrome (POTS), but microneurography is clinically impractical. We investigated whether the Valsalva phase 2 diastolic blood pressure rise (DBP_VM2l_rise_) serves as a sympathetic marker and proposed enhanced neurovascular transduction as a pathophysiological mechanism in hyperadrenergic POTS.

**Methods:**

We included 21 POTS women and 22 healthy women to perform Valsalva and microneurography. MSNA spike rate was obtained using stationary wavelet transformation. The DBP_VM2l_rise_ cut point for hyperadrenergic POTS was optimized by the golden section search with its correlation to phase 2 MSNA spike rate as an objective function. We defined peripheral sympathetic neurovascular transduction (psNVT) as a ratio of DBP_VM2l_rise_ to early phase 2 MSNA increase. We compared Valsalva responses between the identified hyperadrenergic and non-hyperadrenergic POTS.

**Results:**

The DBP_VM2l_rise_ strongly correlated with the Valsalva phase 2 MSNA spike rate percentage change from baseline in healthy (*r* = 0.874, *p* < 0.001). The DBP_VM2l_rise_ cutoff criterion of 15 mmHg optimally separated POTS into 7 hyperadrenergic (≥ 15 mmHg, *r* = 0.902, *p* = 0.014) and 14 non-hyperadrenergic (< 15 mmHg, *r* = 0.629, *p* = 0.021). Although similar MSNA spike rate, the hyperadrenergic group had higher baseline systolic blood pressure (118 ± 10 vs 105 ± 12 mmHg, *p* = 0.026), shorter pressure recovery time (1.15 ± 0.75 vs 2.59 ± 1.17 s, *p* = 0.048), and higher psNVT (2.60 ± 1.02 vs 0.58 ± 0.46 mmHg/spike·s^−1^, *p* < 0.001) than the non-hyperadrenergic POTS.

**Conclusion:**

DBP_VM2l_rise_ ≥ 15 mmHg could be a sympathetic clinical marker and could identify hyperadrenergic POTS, characterized by enhanced neurovascular transduction despite comparable MSNA levels. This novel pathophysiological insight underscores the importance of sympathetic markers in POTS clinical management.

**Supplementary Information:**

The online version contains supplementary material available at 10.1007/s10286-025-01183-z.

## Introduction

Postural tachycardia syndrome (POTS) is a heterogeneous condition associated with a complex pathophysiology. Patients with POTS experience aggravated orthostatic symptoms accompanied by sustained increase in heart rate (HR) at least 30 beats per minute upon standing without orthostatic hypotension [1]. In a subset of these patients, the main pathophysiological mechanism has been proposed to be primary sympathetic activation (hyperadrenergic POTS) [2, 3]. Sympathetic activation can be measured directly using microneurography techniques to obtain muscle sympathetic nerve activity (MSNA). This technique requires highly specialized skills, equipment, and is not available in clinics. Indirect clinical markers of increased sympathetic activity, such as high systolic blood pressure (SBP) overshoot during Valsalva, orthostatic hypertension, high orthostatic plasma catecholamines [4–6], and response to sympatholytic drugs have been proposed [3, 7]. Recently, we showed that a subset of patients with POTS have increased resting MSNA, indicating greater central sympathetic outflow, and that can be identified by an exaggerated diastolic blood pressure (DBP) increase during late phase 2 of the Valsalva maneuver (DBP_VM2l_rise_). This biomarker can also identify those who improved POTS symptoms with central sympatholytic therapy with guanfacine. Taken together, these data are consistent with the generally accepted concept of a hyperadrenergic POTS phenotype driven by central sympathetic activation [8].

We have also encountered patients with POTS who had an exaggerated DBP_VM2l_rise_ even though they had a normal MSNA response, suggesting an enhanced neurovascular translation (an exaggerated vasoconstriction for a given sympathetic activity). We tested this possibility in 23 unselected female patients with POTS and 23 matched controls. We used a wavelet denoising technique to identify individual sympathetic spikes, which allowed us to correlate acute changes in spike rate that occur during the Valsalva maneuver (VM) with corresponding blood pressure changes.

## Methods

### Participants

We recruited 23 female patients with POTS and 23 healthy female subjects aged between 18–55 years old. POTS participants were diagnosed by clinicians specialized in the autonomic field at our Vanderbilt Autonomic Dysfunction Center. All patients with POTS met the following criteria: (1) HR increase at least 30 beats/min within 10 min after standing, (2) no orthostatic hypotension, and (3) chronic orthostatic symptoms for at least 6 months. Subjects stopped any medications affecting the autonomic nervous system for at least five half-lives before studies. A minimal number of psychiatric drugs have been continued as necessary, after consulting with a team of physicians. All participants gave their informed consent.

### Protocol

The study was approved by the Vanderbilt University Institutional Review Board in Human Research and registered at clinicaltrials.gov (NCT04050410). The protocol was conducted at the Vanderbilt Autonomic Dysfunction Center.

On the screening day, all subjects performed an active stand test with intermittent HR and BP measurements at 3, 5, and 10 min after standing. Orthostatic vitals were chosen from the highest upright HR and the average BP between measurements at 3 and 5 min [9]. Supine and upright plasma norepinephrine (NE) levels were collected during the active stand test. On the study day, after subjects were supine for ≥ 30 min before studies to allow fluid redistribution, blood volume was measured by the carbon monoxide rebreathing method. Blood volume deviation (BV_deviation_) as a percentage of normal values was calculated based on weight, height, and sex [10]. The autonomic function tests were started after at least 30 min of being supine. Subjects were asked to breathe normally for 5 min for baseline recordings. After this, subjects performed a training Valsalva prior to the Valsalva maneuver for analysis. In each Valsalva, subjects were asked to relax for 1 min, then blow into a 10-mL syringe with a tiny leak (22G hole) at a pressure of 30 mmHg for 15 s followed by a quiet and relaxing period of one minute. The leak hole served to maintain a connection between intrathoracic pressure and a manometer [11, 12]. Both the researcher and the subject could monitor the straining pressure in real time on a tablet display.

### Data recording

Microneurography of the left peroneal nerve was performed using a unipolar tungsten electrode (2 MOhms, FCH, Inc, Bowdoinham, ME). MSNA was filtered (300 Hz–5 kHz) and amplified (gain range 50 V and resolution 25 nV) with a stage amplifier (Neuroamp EX, ADInstruments Inc, Colorado Springs CO). Satisfactory recordings of MSNA were defined by (1) heart pulse synchronicity; (2) facilitation during Valsalva straining and suppression during the hypertensive overshoot phase after release; and (3) no changes during tactile or auditory stimulation.

Electrocardiogram (ECG, Ivy 450C, Ivy Biomedical Systems, Inc, Brandford CT, USA), continuous finger blood pressure (Finapres NOVA, the Netherlands), segmental body impedance (BIM, Diefenbach GmbH, Germany), and MSNA were recorded with Powerlab 16/35 and Labchart 8 (ADInstruments Inc, Colorado Springs CO). The sample rates were 20 kHz for MSNA and 1 kHz for ECG and blood pressure. Finger blood pressure values were cross-calibrated with oscillometric brachial blood pressure arm cuff measurements during baseline (Ivy 450C, Ivy Biomedical Systems, Inc, Brandford CT, USA).

### Data processing and analysis

Data were processed with our customized MATLAB software (Physiowave^©^, A. Diedrich, Vanderbilt University Medical Center, TN, USA). Fiducial points in ECG and continuous blood pressure waveforms were detected and visually verified. Beat-to-beat time series were validated, interpolated, and resampled at 5 Hz. Stroke volume (SV), cardiac output (CO), and total peripheral resistance (TPR) were derived from blood pressure waveforms (Modelflow®, Finapres Medical System BV, the Netherlands) [13]. Baseline values were averaged over the 5-min resting period. Power spectral analysis was performed using a fast Fourier transform (FFT)-based Welch’s method [14].

MSNA spikes were denoised and detected by the stationary wavelet transformation with a two-stage kurtosis method [15]. Spike rate was estimated by convolving delta functions at spike locations with a 3-Hz cutoff frequency Gaussian filter [16]. Additionally, beat-to-beat mean spike rate was calculated by averaging the spike rate for each R–R interval. Burst rate and burst incidence were derived from the integrated MSNA (resistance–capacitance low-pass filter with a time constant of 0.1 s) during resting supine baseline [17, 18].

**Fig. 1:**
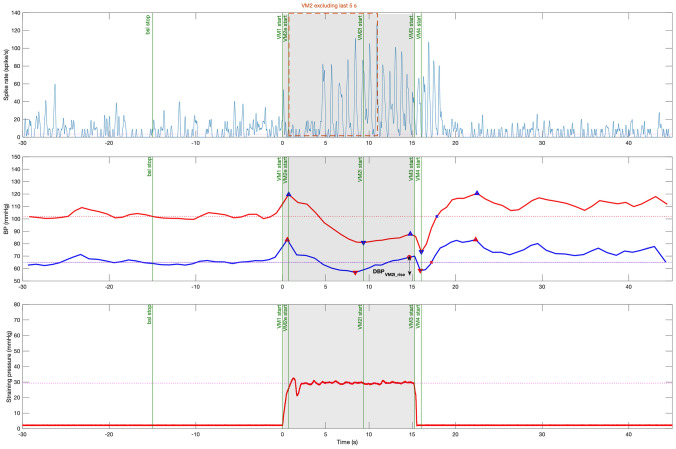
Example recording of a healthy subject performing a 30-mmHg Valsalva maneuver. Valsalva phases were defined based on straining time and blood pressure changes (green vertical lines). *Bsl* baseline, *VM1* Valsalva phase 1, *VM2e* early Valsalva phase 2, *VM2l* late Valsalva phase 2, *VM3* Valsalva phase 3, *VM4* Valsalva phase 4. Top panel: continuous MSNA spike rate smoothed by a 3-Hz cutoff frequency Gaussian filter. The orange box represents MSNA spike rate in phase 2 excluding the last 5 s. Middle panel: continuous finger systolic blood pressure (SBP, red) and diastolic blood pressure (DBP, blue). Horizontal lines represent baseline blood pressures. The late phase 2 DBP rise (DBP_VM2l_rise_) is the difference between the VM2 DBP nadir and the subsequent maximum at the end of VM2 (vertical line with arrows). Bottom panel: Valsalva straining pressure. The subject was asked to hold pressure at 30 mmHg for 15 s (gray area)

Following common Valsalva maneuver phases were defined and analyzed: Valsalva baseline phase from 45 to 15 s prior to straining. Phase 1 (VM1) begins at the onset of straining. Phase 2 (VM2) starts from the maximum blood pressure until the end of straining. VM2 was divided into early (VM2e) and late (VM2l) phases at the nadir of SBP. Phase 3 (VM3) starts after the release of the strain and ends at the minimum blood pressure. Finally, phase 4 (VM4) is the period of blood pressure recovery to baseline level, overshoot, and return to baseline level (Fig. 1). The late phase 2 DBP rise (DBP_VM2l_rise_) is defined as the difference between the VM2 DBP nadir and the subsequent maximum at the end of VM2. We chose to analyze changes in DBP because it has been shown to correlate strongly with MSNA, unlike SBP [18].

MSNA spike rate of each phase was analyzed in absolute values and delta changes from Valsalva baseline level. The delta change values were also expressed as percentage changes from the baseline phase of the Valsalva. When studying the association between MSNA and blood pressure changes in Valsalva phase 2, we excluded the last 5 s of MSNA activity to account for the 5 s delay on blood pressure response. In other words, MSNA during the last 5 s of phase 2 would not affect the blood pressure values at the end of phase 2 (Fig. 1, orange box) [19]. We defined central sympathetic baroreflex sensitivity ($\mathrm{csBRS}$) as the ratio between the delta change in spike rate and the preceding delta change in diastolic blood pressure within the early phase 2 (Eq. 1a). In addition, peripheral sympathetic neurovascular transduction (psNVT) was defined as the ratio between diastolic blood pressure rise during late phase 2 and the preceding spike rate change in early phase 2 (Eq. 1b).

Equation 1a, 1b central sympathetic baroreflex sensitivity and peripheral sympathetic neurovascular transduction.

1a $$\mathrm{csBRS}= \frac{\text{mean VM}2\text{e spike rate}-\text{mean baseline spike rate}}{\text{mean baseline DBP}-\text{minimum VM}2\text{e DBP}}$$

1b $$\mathrm{psNVT}= \frac{\text{maximum VM}2\text{l DBP}-\text{minimum VM}2\text{e DBP}}{\text{mean VM}2\text{e spike rate}-\text{mean baseline spike rate}}$$

csBRS = central sympathetic baroreflex sensitivity (spike·s^−1^/mmHg).

psNVT = peripheral sympathetic neurovascular transduction (mmHg/spike·s^−1^).

VM2e = early phase 2 of Valsalva maneuver.

VM2l = late phase 2 of Valsalva maneuver.

DBP = diastolic blood pressure (mmHg).

### Cut point search for DBP rise (DBP_VM2l_rise_)

Our primary objective was to find the optimal cut point value for DBP_VM2l_rise_ to distinguish hyperadrenergic from non-hyperadrenergic POTS. We defined the optimal DBP_VM2l_rise_ cut point as the value where the highest correlations between MSNA and DBP_VM2l_rise_ were found in both hyperadrenergic (above the cut point value) and non-hyperadrenergic POTS (below the cut point value) using the golden section search method, as detailed in Appendix 1 [20].

### Statistical analysis

Data were presented as mean ± standard deviation (SD) unless otherwise specified. Comparisons between healthy subjects and patients with POTS were performed using an unpaired *t* test for normally distributed data or the Wilcoxon rank-sum test for non-normally distributed data. Data normality was checked by the Anderson–Darling test. Correlation analyses were done using Pearson’s linear correlation. Differences in selected Valsalva metrics that may be influenced by different baseline hemodynamics, when applicable, were corrected by analysis of covariance (ANCOVA). Statistical analyses were performed in MATLAB (The MathWorks, Inc., Natick, MA). Two-tailed *p* values ≤ 0.05 were considered statistically significant.

The current study focused on sympathetic function comparisons in patients with POTS. Multiple comparisons with adjusted* p* values in following sympathetic markers will be further computed by the Holm–Bonferroni method to control the family-wise error rate of sympathetic function [21]: Valsalva baseline SBP, Valsalva baseline MSNA spike rate, low frequency power of SBP (logLF_SBP_), pressure recovery time (PRT), SBP overshoot, csBRS, and psNVT. Other variables were considered exploratory and were not adjusted for multiplicity.

## Results

### Participants, general characteristics, and normal breathing hemodynamics

One healthy subject and two patients with POTS were excluded due to arrhythmia and poor Valsalva straining quality. In total, 22 healthy subjects and 21 patients with POTS were included in the analysis. Out of 21 patients with POTS, only 19 subjects had valid MSNA recordings. Patients with POTS and controls were similar in age, weight, and height, but patients with POTS had more blood volume deficit (Table 1, *p* < 0.001), higher orthostatic HR increase (*p* < 0.001), higher supine resting HR (Table 2; *p* < 0.001), higher SBP (*p* = 0.011), higher DBP (*p* = 0.024). Patients with POTS had significantly lower root mean square of successive differences between heartbeats (RMSSD), a time domain parameter of cardiovagal modulation (*p* = 0.015). Similarly, the spectral analysis index of cardiovagal modulation (logHF_RRI_) tended to be lower in POTS, but it did not reach statistical significance (*p* = 0.077). Other spectral analysis parameters, as well as resting MSNA burst rate and burst incidence were not different between the two groups (Table 2).

**Table 1:** General characteristics and blood volume status in female healthy controls and patients with POTS

	Healthy	POTS	*p* value
General characteristics	(*n* = 22)	(*n* = 21)	
Age (year)	32 ± 9	28 ± 7	0.559
Weight (kg)	63.4 ± 9.0	67.9 ± 11.0	0.302
Height (cm)	166 ± 7	169 ± 6	0.236
Orthostatic vitals	(*n* = 21)	(*n* = 21)	
deltaHR (beat/min)	26 ± 15	45 ± 19	< 0.001
deltaSBP (mmHg)	2 ± 9	5 ± 7	0.171
deltaDBP (mmHg)	7 ± 7	10 ± 8	0.162
Orthostatic NE	(*n* = 22)	(*n* = 19)	
Supine NE (pg/mL)	217 ± 97	248 ± 123	0.367
Upright NE (pg/mL)	478 ± 243 (*n* = 20)	578 ± 318	0.361
Blood volume measurements	(*n* = 16)	(*n* = 19)	
Hematocrit (Hct, %)	37.32 ± 2.17	38.87 ± 1.98	0.034
Hemoglobin concentration (Hb, g/dL)	12.41 ± 1.11	13.04 ± 0.65	0.071
Blood volume (BV, mL/kg)	74.84 ± 9.12	64.55 ± 10.34	0.004
Blood volume deviation (BV_deviation_, %)	− 1.40 ± 10.10	− 14.40 ± 10.74	< 0.001

Values presented as mean ± SD, BV_deviation_ is a percentage difference between measured blood volume and normal blood volume predicted from sex, weight, and height [10]. Orthostatic vitals presented as standing minus supine HR difference (deltaHR), systolic blood pressure difference (deltaSBP), and diastolic blood pressure difference (deltaDBP)

*NE* plasma norepinephrine concentration

**Table 2:** Spectral powers and hemodynamics of 5-min supine normal breathing in female healthy controls and patients with POTS

	Healthy (*n* = 22)	POTS (*n* = 21)	*p* value
HR (beat/min)	70 ± 9	85 ± 15	< 0.001
SBP (mmHg)	104 ± 9	113 ± 12	0.011
DBP (mmHg)	67 ± 6	71 ± 8	0.027
SV (mL)	85 ± 16 (*n* = 21)	86 ± 13 (*n* = 21)	0.808
CO (L/min)	5.83 ± 1.08 (*n* = 21)	7.21 ± 1.45 (*n* = 21)	0.001
TPR (dyn·s/cm^5^)	1176 ± 278 (*n* = 21)	1003 ± 189 (*n* = 21)	0.029
RMSSD (ms)	47 ± 25	33 ± 33	0.015
logLF_RRI_	2.86 ± 0.43	2.70 ± 0.40	0.208
logHF_RRI_	2.72 ± 0.47	2.40 ± 0.67	0.077
logLF_SBP_	0.57 ± 0.35	0.78 ± 0.42	0.089
MSNA burst rate (burst/min)	11.05 ± 6.56 (*n* = 16)	11.49 ± 6.16 (*n* = 19)	0.838
MSNA burst incidence (burst/100beats)	16.07 ± 10.48 (*n* = 16)	14.00 ± 8.38 (*n* = 19)	0.519

Values presented as mean ± SD

*HR* heart rate, *SBP* systolic blood pressure, *DBP* diastolic blood pressure, *SV* stroke volume, *CO* cardiac output, *TPR* total peripheral resistance, *RMSSD* root mean square of successive differences between normal heartbeats, *LF* low frequency power, *HF* high frequency power, *RRI* R–R interval of an electrocardiogram, *log* base 10 logarithm, *MSNA* muscle sympathetic nerve activity

### Heart rate, blood pressure, and MSNA spike rate during Valsalva maneuver

**Fig. 2:**
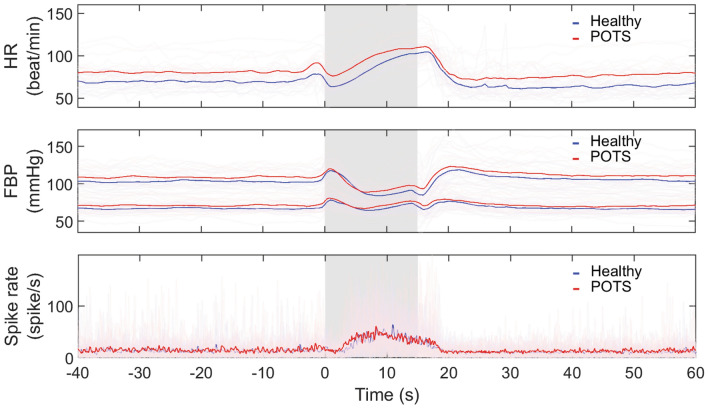
Overlay plots of heart rate (HR, top, healthy *n* = 22, POTS *n* = 21), finger blood pressure (FBP, middle, healthy *n* = 22, POTS *n* = 21) where upper line represents systolic blood pressure and lower line represents diastolic blood pressure, and MSNA spike rate (bottom, healthy *n* = 17, POTS *n* = 19) changes during Valsalva maneuver. Light lines represent individual responses. Dark lines are groups’ average. Gray areas show 30-mmHg Valsalva straining period for 15 s

Patients with POTS had significantly higher HR than healthy subjects during baseline (POTS vs healthy, 81 ± 14 vs 70 ± 9 beat/min, *p* = 0.005) and early phase 2 of Valsalva (101 ± 16 vs 90 ± 17 beat/min, *p* = 0.041). Blood pressure responses were generally comparable throughout the Valsalva maneuver. MSNA spike rates were not different between the two groups both during baseline (14.28 ± 6.38 vs 13.36 ± 6.37 spike/s, *p* = 0.670) and early phase 2 of Valsalva (32.83 ± 15.41 vs 29.55 ± 11.62 spike/s, *p* = 0.680) (Fig. 2).

Valsalva sympathetic markers including SBP overshoot (19 ± 11 vs 20 ± 15 mmHg, *p* = 0.714), PRT (2.11 ± 1.24 vs 3.10 ± 5.62 s, *p* = 0.932), and DBP_VM2l_rise_ (13 ± 10 vs 13 ± 10 mmHg, *p* = 0.961), as well as csBRS (2.91 ± 3.91 vs 3.05 ± 5.66 spike·s^−1^/mmHg, *p* = 0.590) and psNVT (1.22 ± 1.16 vs 1.07 ± 0.76 mmHg/spike·s^−1^, *p* = 0.899) were not different between POTS and healthy. Valsalva ratio was also similar between the two groups (1.86 ± 0.41 vs 1.98 ± 0.32, *p* = 0.287).

### Correlations between Valsalva phase 2 MSNA and DBP_VM2l_rise_

**Fig. 3:**
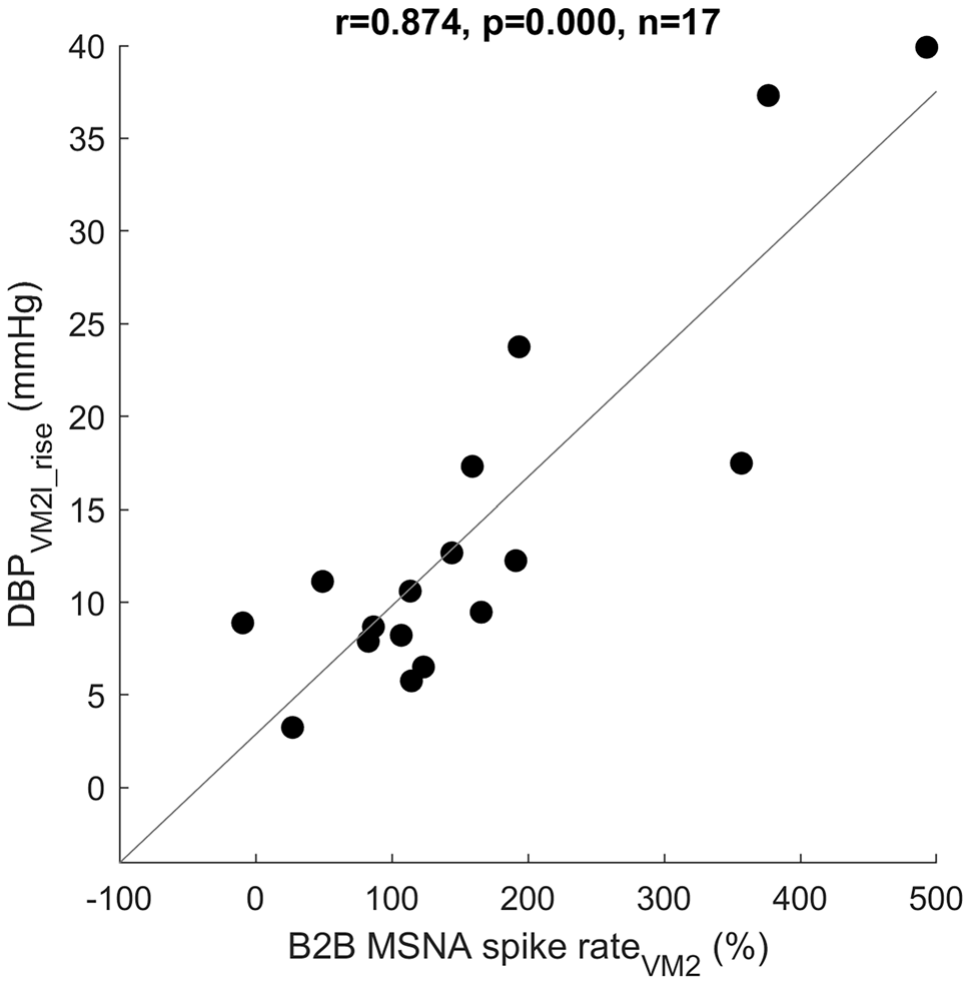
Correlation between beat-to-beat mean spike rate change during phase 2 except last 5 s in percent from baseline (B2B MSNA spike rate_VM2_) and diastolic blood pressure rise during late phase 2 (DBP_VM2l_rise_) in healthy control (*r* = 0.874, *p* < 0.001)

**Fig. 4:**
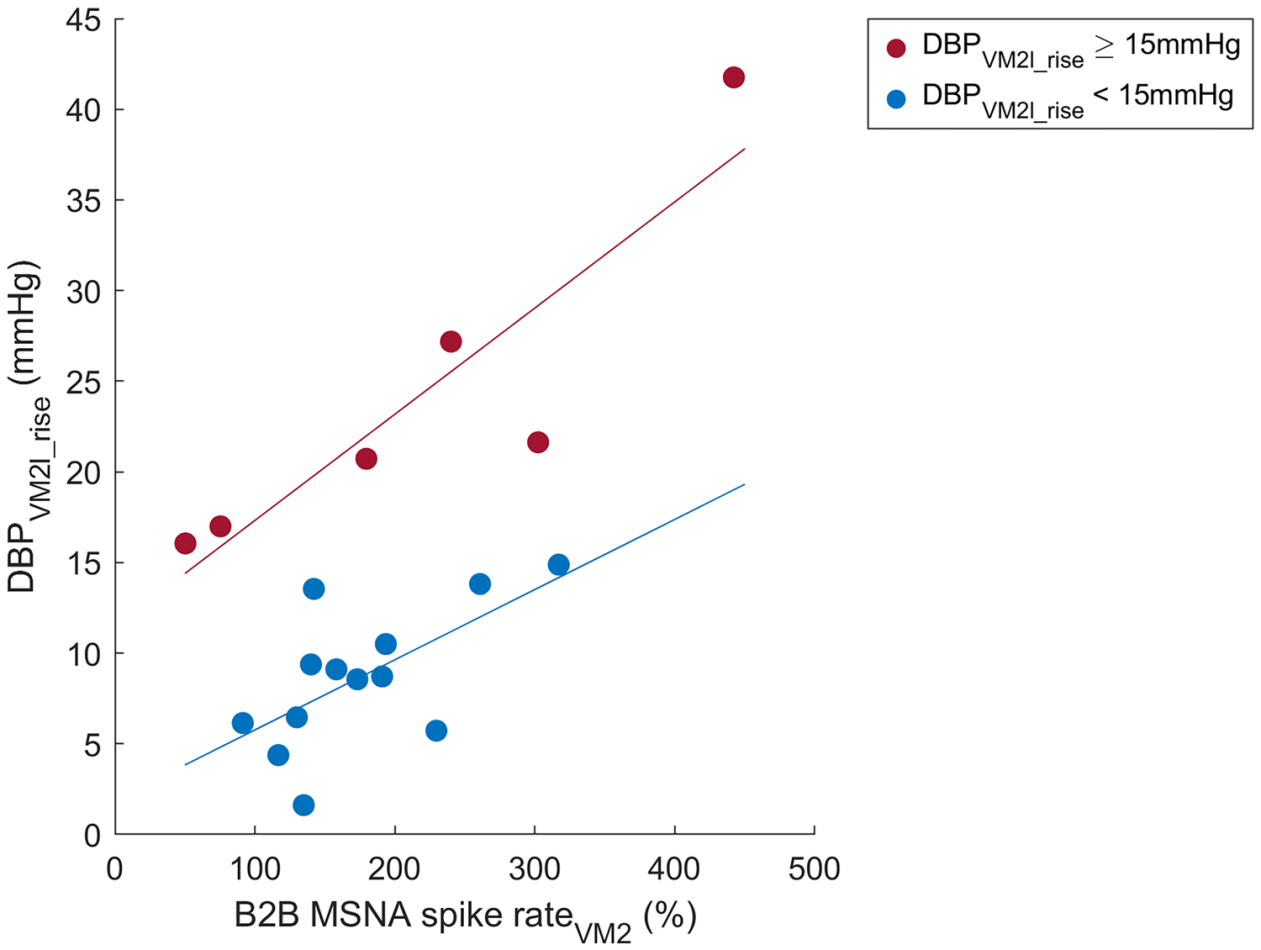
Correlation analyses between beat-to-beat mean MSNA spike rate percentage difference from baseline during phase 2, excluding last 5 s (B2B MSNA spike rate_VM2_) and late phase 2 DBP rise (DBP_VM2l_rise_) in patients with POTS having DBP_VM2l_rise_ ≥ 15 mmHg (*r* = 0.902, *p* = 0.014, *n* = 6, red) and in patients with POTS having DBP_VM2l_rise_ < 15 mmHg (*r* = 0.629, *p* = 0.021, *n* = 13, blue)

We found a strong correlation between the phase 2 beat-to-beat mean spike rate percentage change from baseline (B2B MSNA spike rate_VM2_) and the DBP_VM2l_rise_ in healthy subjects (*r* = 0.874, *p* < 0.001; Fig. 3). The correlation was weaker in POTS (*r* = 0.661, *p* = 0.002) due to possibly greater heterogeneity (Fig. 4). Absolute baseline and phase 2 spike rate were not significantly correlated with DBP_VM2l_rise_.

### Golden section search for DBP_VM2l_rise_ optimal cut point

Figure 4 shows two possible clusters of patients with POTS with good correlations between MSNA and DBP changes but with different offsets. To find the optimal cut point of DBP_VM2l_rise_ between the two clusters in POTS, we maximized the product of the correlation coefficients of each cluster by varying the cut point value using the golden section search method in nineteen patients with POTS who had valid MSNA recording (Appendix 1). After 13 iterations, a DBP_VM2l_rise_ cut point of 14.93 mmHg yielded the highest product of the correlation coefficients of the cluster above and below the cut point within the tolerance of 0.1 mmHg. The final optimized DBP_VM2l_rise_ cut point separated all patients with POTS into two groups, DBP_VM2l_rise_ ≥ 15 mmHg (*n* = 7) and DBP_VM2l_rise_ < 15 mmHg (*n* = 14). Both groups had significant correlation between their DBP_VM2l_rise_ and the B2B MSNA spike rate_VM2_ at *r* = 0.902 (*p* = 0.014) for the former group, and *r* = 0.629 (*p* = 0.021) for the latter group (Fig. 4).

### Comparison between POTS groups with DBP_VM2l_rise_ ≥ 15 versus < 15 mmHg

All patients with POTS in the DBP_VM2l_rise_ ≥ 15 mmHg group had reported at least one hyperadrenergic symptom on the pre-screening questionnaire. The proportions of patients experiencing palpitation, profuse sweating, and flushing episodes were 86%, 71%, and 57%, respectively. There were no differences in age, orthostatic BP, orthostatic NE, and blood volume status between POTS groups with DBP_VM2l_rise_ ≥ 15 versus < 15 mmHg (Table 3).

**Table 3:** General characteristics of POTS having DBP_VM2l_rise_ < 15 mmHg and ≥ 15 mmHg

	POTS DBP_VM2l_rise_ < 15 (*n* = 14)	POTS DBP_VM2l_rise_ ≥ 15 (*n* = 7)	*p* value
General characteristics			
Age (year)	28 ± 8	29 ± 7	0.921
Weight (kg)	65 ± 7	75 ± 14	0.045
Orthostatic vitals			
deltaHR (beat/min)	51 ± 19	33 ± 12	0.041
deltaSBP (mmHg)	5 ± 6	4 ± 9	0.703
deltaDBP (mmHg)	9 ± 7	12 ± 10	0.555
Orthostatic NE			
Supine NE (pg/mL)	261 ± 134 (*n* = 12)	227 ± 107	0.570
Upright NE (pg/mL)	550 ± 307 (*n* = 12)	626 ± 354	0.629
Blood volume measurements			
Blood volume deviation (BV_deviation_, %)	− 13.58 ± 10.37 (*n* = 12)	− 15.80 ± 12.05	0.773

Values presented as mean ± SD, BV_deviation_ is a percentage difference between measured blood volume and normal blood volume predicted from sex, weight, and height [10]; Orthostatic vitals presented as standing minus supine heart rate difference (deltaHR), systolic blood pressure difference (deltaSBP), and diastolic blood pressure difference (deltaDBP)

*NE* plasma norepinephrine concentration

**Fig. 5:**
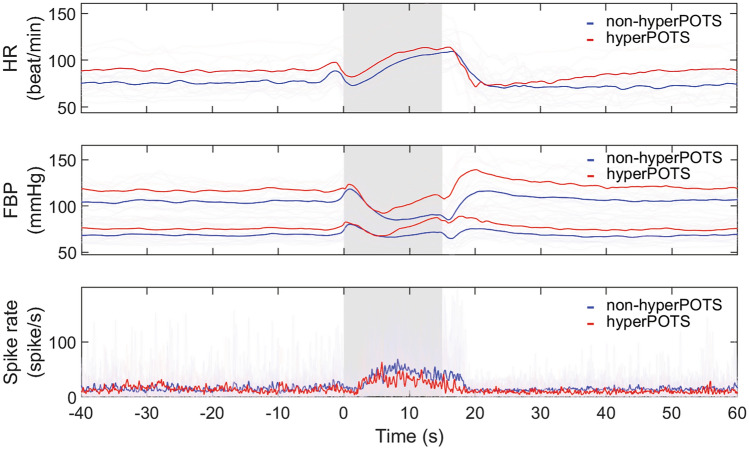
Overlay plots of hyperadrenergic POTS (hyperPOTS, red) and non-hyperadrenergic POTS (non-hyperPOTS, blue) of heart rate (HR, top, non-hyperPOTS *n* = 14, hyperPOTS *n* = 7), finger blood pressure (FBP, middle, non-hyperPOTS *n* = 14, hyperPOTS *n* = 7) where upper line represents systolic blood pressure and lower line represents diastolic blood pressure, and MSNA spike rate (bottom, non-hyperPOTS *n* = 13, hyperPOTS *n* = 6) changes during Valsalva maneuver. Light lines represent individual responses. Dark lines are groups’ average. Gray areas show 30-mmHg Valsalva straining period for 15 s

At the baseline phase of Valsalva, the POTS group with DBP_VM2l_rise_ ≥ 15 mmHg (*n* = 7) had higher SBP (118 ± 10 vs 105 ± 12 mmHg, *p* = 0.026) and HR (90 ± 19 vs 76 ± 10 beat/min, *p* = 0.038) than those with DBP_VM2l_rise_ < 15 mmHg (*n* = 14). The high DBP_VM2l_rise_ group continued to exhibit higher systolic blood pressure than the other patients with POTS throughout the end of late phase 2 (SBP_endVM2l_, 116 ± 17 vs 93 ± 15 mmHg, *p* = 0.005), and peak of phase 4 (SBP_maxVM4_, 143 ± 21 vs 120 ± 17 mmHg, *p* = 0.019) of the Valsalva, while the SBP drop in early phase 2 was similar (SBP_VM2e_deltabsl_, − 27 ± 9 vs − 22 ± 9 mmHg, 0.200). Interestingly, the duration of early phase 2 (the point of SBP nadir) was shorter in the DBP_VM2l_rise_ ≥ 15 mmHg group (Duration_VM2e_, 5.57 ± 1.93 vs 8.28 ± 2.59 s, *p* = 0.025) (Fig. 5). However, these Valsalva BP metrics were not statistically different by group effect after we corrected using ANCOVA with baseline SBP as a covariate; SBP_endVM2l_(*F*(1,18) = 2.986, *p* = 0.101), SBP_maxVM4_(*F*(1,18) = 0.660, *p* = 0.427), SBP_VM2e_deltabsl_(*F*(1,18) = 0.048, *p* = 0.828), and Duration_VM2e_(*F*(1,18) = 2.023, *p* = 0.172).

Absolute MSNA spike rate of both groups was not different during baseline (13.45 ± 7.08 vs 14.66 ± 6.30 spike/s, *p* = 0.714) and early phase 2 (24.83 ± 7.23 vs 36.52 ± 16.97 spike/s, *p* = 0.127) (Fig. 5). However, the high DBP_VM2l_rise_ group had greater BP responses with respect to the activated MSNA spike rate as evidenced by higher psNVT than the other group (2.60 ± 1.02 vs 0.58 ± 0.46 mmHg/spike·s^−1^, *p* < 0.001), while the csBRS was not significantly different (0.62 ± 2.69 vs 3.96 ± 4.01 spike·s^−1^/mmHg, *p* = 0.084). The PRT was lower (1.15 ± 0.75 vs 2.59 ± 1.17 s, *p* = 0.005) and the SBP overshoot tended to be higher (25 ± 12 vs 15 ± 10 mmHg, *p* = 0.079) in the high DBP_VM2l_rise_ group. The ANCOVA analysis confirmed the group effect differences in psNVT (*F*(1,16) = 9.146, *p* = < 0.001), and PRT (*F*(1,18) = 5.044, *p* = 0.048) after adjusting for differences in baseline SBP (Table 4). The DBP_VM2l_rise_ was still significantly different between the two POTS groups, even after correction for differences in baseline SBP (F(1, 18) = 18.726, *p* < 0.001).

**Table 4:** Valsalva sympathetic metrics of POTS having DBPVM2l_rise < 15 mmHg and ≥ 15 mmHg

	POTS DBP_VM2l_rise_ < 15 (*n* = 14)	POTS DBP_VM2l_rise_ ≥ 15 (*n* = 7)	*p* value*	Adjusted *p* value^#^
Pressure recovery time (s)	2.59 ± 1.17	1.15 ± 0.75	0.048	0.238
SBP overshoot (mmHg)	15 ± 10	25 ± 12	0.458	0.915
csBRS (spike·s^−1^/mmHg)	3.96 ± 4.01 (*n* = 13)	0.62 ± 2.69 (*n* = 6)	0.327	0.980
psNVT (mmHg/spike·s^−1^)	0.58 ± 0.46 (*n* = 13)	2.60 ± 1.02 (*n* = 6)	< 0.001	0.003

Values presented as mean ± SD

*csBRS* central sympathetic baroreflex sensitivity,* psNVT* peripheral sympathetic neurovascular transduction.

*p values were corrected for different Valsalva baseline SBP by ANCOVA

^#^Adjusted *p* values were *p* values corrected for group differences of seven multiple comparisons by the Holm–Bonferroni method

To evaluate sympathetic function differences between POTS groups with DBP_VM2l_rise_ ≥ 15 versus < 15 mmHg, the Holm–Bonferroni method was applied to sequentially adjust *p* values across seven sympathetic outcomes [21]. After correction, psNVT remained significantly higher in the DBP_VM2l rise_ ≥ 15 mmHg group (adjusted *p* = 0.003). Other sympathetic outcomes including csBRS (adjusted *p* = 0.980), Valsalva baseline SBP (adjusted *p* = 0.156), Valsalva baseline MSNA spike rate (adjusted *p* = 0.806), logLF_SBP_ (adjusted *p* = 0.446), PRT (adjusted *p* = 0.238), and SBP overshoot (adjusted *p* = 0.915) were not statistically significant. Subsequently, we further compute 95% confidence interval (CI) of the main group difference variable, psNVT. The 95% CI of the difference in psNVT was [1.155, 2.867].

### Correlation between DBP_VM2l_rise_ and selected sympathetic-related markers in POTS

**Fig. 6:**
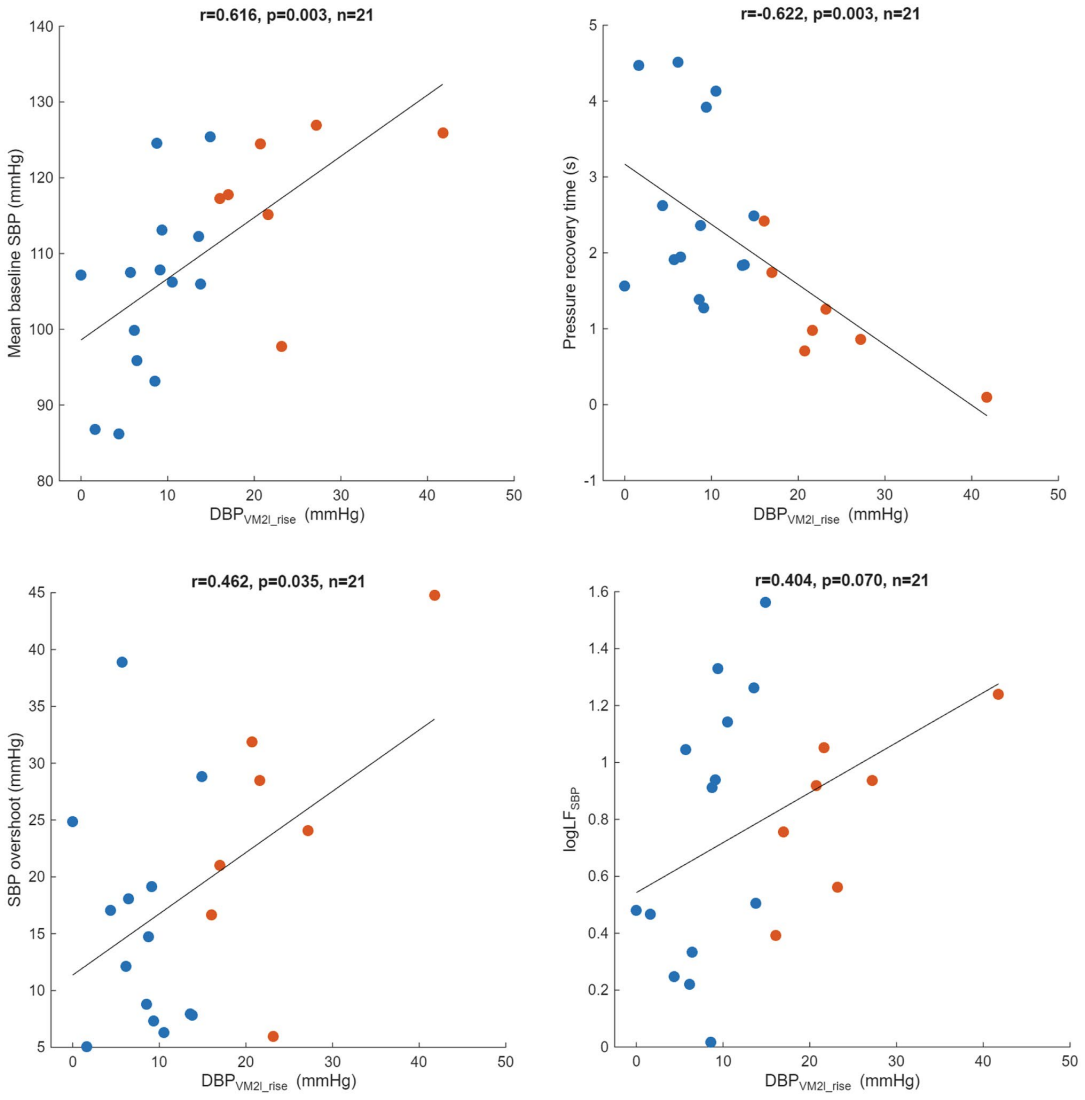
Correlation analyses between late phase 2 DBP rise (DBP_VM2l_rise_) and sympathetic-related markers including mean baseline SBP (upper left, *r* = 0.616, *p* = 0.003), pressure recovery time (upper right, *r* = − 0.622, *p* = 0.003), SBP overshoot (lower left, *r* = 0.462, *p* = 0.035), and logarithm of low frequency power of resting SBP variability (logLF_SBP_, lower right, *r* = 0.404, *p* = 0.070) in POTS having DBP_VM2l_rise_ < 15 mmHg (blue) and ≥ 15 mmHg (red)

DBP_VM2l_rise_ was significantly correlated with Valsalva baseline SBP (*r* = 0.616, *p* = 0.003), pressure recovery time (PRT, *r* = − 0.622, *p* = 0.003), and SBP overshoot (*r* = 0.462, *p* = 0.035). DBP_VM2l_rise_ did not correlate with the low frequency power of SBP during resting (logLF_SBP_, *r* = 0.404, *p* = 0.070, Fig. 6). No correlation was found between the DBP_VM2l_rise_ and blood volume deviation (*r* = − 0.280, *p* = 0.246).

## Discussion

We previously reported a subset of patients with POTS characterized by augmented central sympathetic outflow, as assessed by increased resting sympathetic nerve traffic (MSNA), and associated with greater diastolic blood pressure response to phase 2 of the VM (DBP_VM2l_rise_) [8]. This central hyperadrenergic phenotype was accompanied by increased resting blood pressure and an increase in upright BP. Furthermore, patients identified using the DBP_VM2l_rise_ clinical biomarker reported clinical improvements after treatment with the central sympatholytic guanfacine, consistent with the hypothesis that this is a central hyperadrenergic POTS phenotype.

Here we report a different cohort of patients with POTS who also have an exaggerated diastolic pressor response to phase 2 of the VM, but are associated with augmented neurovascular coupling rather than an increase in resting central sympathetic outflow. We used a novel wavelet denoising technique to identify individual sympathetic action potential spikes, which allowed us to correlate acute changes in spike rate that occur during the VM with corresponding blood pressure changes. Such time resolution was not previously possible using the standard integrated MSNA signal. This subset of patients with POTS had a greater increase in diastolic blood pressure in response to phase 2 of the VM, despite a similar MSNA spike rate, i.e. these patients are characterized by augmented neurovascular transduction (NVT). Our preliminary results suggest that moxonidine selectively suppresses sympathetic Valsalva responses including the DBP_VM2l_rise_ in hyperadrenergic POTS but not in non-hyperadrenergic patients (Appendix 2). This conclusion is based on a limited sample size, but it is in agreement with the concept that the enhanced vasoconstriction is sympathetically driven. These patients also had higher baseline SBP, and shorter pressure recovery time during phase 4 of the VM. These findings are consistent with high sympathetic activity. We confirmed by ANCOVA that the higher DBP_VM2l_rise_ and shorter PRT were not just consequences of higher baseline blood pressure offsets in the hyperadrenergic group.

Although the DBP_VM2l_rise_ was correlated with MSNA percentage changes, its correlation to actual phase 2 MSNA spike rate was not significant. These results suggest that vasoconstriction occurs in response to relative changes in MSNA, rather than to absolute neural activity. Calculating percentage changes in MSNA corrects for the interindividual variation in MSNA, which makes it uncorrelated with baseline BP and HR [22–24]. Our findings are in agreement with previous work by Schrezenmaier et al., which showed a strong correlation between the percentage change of MSNA in early phase 2 and the SBP change in late phase 2 [25]. The strong correlation of beat-to-beat MSNA spike rate_VM2_, which excludes the last 5 s of phase 2 MSNA to account for the rise in late phase 2 DBP, supports a theory of a 5-s feedforward delay of the neurovascular response [19, 26]. We decided to use the average beat-to-beat spike rate rather than the simple mean spike rate because of the pulse-synchronicity nature of the MSNA firing [23].

We found that DBP_VM2l_rise_ at or above 15 mmHg was an optimal cut point to distinguish hyperadrenergic POTS due to enhanced NVT from non-hyperadrenergic subtypes. This was determined using the golden section search nonlinear optimization method, because it does not require derivation and provides good convergence. We selected the product of correlation coefficients between the DBP_VM2l_rise_ and MSNA as an objective function to ensure that the resulting clusters were physiologically meaningful. Conventional clustering methods, such as k-means clustering, might produce separate data clusters while ignoring the physiological relationship between DBP and MSNA. The search converged in just 13 iterations, suggesting that the 15-mmHg DBP_VM2l_rise_ is the best cut point to separate POTS into two groups based on the correlations with the B2B MSNA spike rate_VM2_ (Fig. 4). The DBP_VM2l_rise_ cutoff criterion that identifies hyperadrenergic POTS associated with enhanced NVT (15 mmHg) is similar to the one we previously found to identify patients with hyperadrenergic POTS due to central sympathetic activation (17 mmHg). These are remarkably close considering that these were determined in different patient cohorts using independent methods, and are within the measurement accuracy.

It is clinically relevant that DBP_VM2l_rise_, a readily available physiological biomarker, appears to accurately identify both hyperadrenergic phenotypes. If validated, it will improve our ability to identify patients with hyperadrenergic POTS. Heretofore, an upright plasma NE ≥ 600 pg/mL has been used as evidence of hyperadrenergic POTS. We were among the first to introduce this threshold in the literature [27], but cautioned that a high plasma NE could be due not only to a primary hyperadrenergic state but could also reflect a compensatory sympathetic activation secondary to a partial neuropathy or volume depletion [27], a concern shared by others [28]. Indeed, plasma NE ≥ 600 pg/mL has been documented in healthy subjects with volume depletion [29], in patients with diabetic neuropathy [30], and even in patients with neuropathic orthostatic hypotension [31]. Furthermore, plasma NE concentration depends on both spillover and clearance [32, 33]. Indeed, the average upright NE was ≥ 600 pg/mL in this patient cohort (Table 3) without differences in between hyperadrenergic and non-hyperadrenergic POTS subgroups. We argue, therefore, that the DBP_VM2l_rise_, which reflects sympathetically mediated vasoconstriction, is more relevant indicator of hyperadrenergic activity and one that can be obtained readily in most autonomic centers, without the need to measure sympathetic nerve traffic or to calculate NVT. Validation of this concept, as well as determining the precise DBP cutoff value will likely require a prospective randomized clinical trial to determine if sympatholytics provide clinical improvement in patients identified using this biomarker. The strength of such an approach is that non-hyperadrenergic patients would likely worsen if treated with a sympatholytic.

We propose a novel pathophysiologic mechanism of hyperadrenergic POTS, driven by enhanced neurovascular coupling instead of increased central sympathetic tone. These patients could generate higher Valsalva DBP response and exhibit high sympathetic features such as shorter PRT, despite the same level of MSNA activation compared to other patients with POTS. We identified this subset of POTS based on their hyperadrenergic responses resulting from augmented neurovascular transduction, which was distinct from patients with POTS with high resting sympathetic discharge, which we have proposed earlier [8]. The proposed mechanism was confirmed by the higher peripheral sympathetic neurovascular transduction (psNVT) found in the hyperadrenergic group (Table 4). The difference in psNVT remained following correction for different baseline SBP between the two POTS groups by ANCOVA and after adjusting for multiple comparisons by the Holm–Bonferroni method. We could conclude that there is a true difference in sympathetic function between hyperadrenergic and non-hyperadrenergic POTS, driven by the psNVT. Factors affecting sympathetic neurovascular transduction may include nitric oxide-mediated dilatation, neural firing pattern, neurotransmitter reuptake function, and sensitivity or density of vascular adrenergic receptors [34]. The mechanism of enhanced transduction is beyond the scope of the current study and warrants for further investigations.

Our preliminary results suggest that moxonidine could selectively suppress sympathetic Valsalva responses including the DBP_VM2l_rise_ in hyperadrenergic POTS, even with a limited sample size (Appendix 2). This emphasizes the importance of subtyping with clinical markers. The central sympatholytic drug, e.g., guanfacine, has been shown to improve symptoms in hyperadrenergic POTS [8]. Moreover, blood volume deficit is a common pathophysiology in POTS [10]. We found that our defined hyperadrenergic POTS and other patients with POTS have similar degrees of blood volume deficit (Table 3). The hypovolemia does not argue against our subtyping of hyperadrenergic POTS and the high blood pressure found in them, since there is an inverse relationship between blood volume and blood pressure in orthostatic intolerance including patients with hyperadrenergic POTS [35]. Chronic use of sympatholytics may improve volume deficits in this patient group since sympathetic overactivity could lead to volume depletion. To the best of our knowledge, no long-term clinical trials have investigated the effects of sympatholytic drugs in patients with hyperadrenergic POTS [36]. A clinical trial of moxonidine with larger sample size and longer duration would be needed to clarify these associations.

## Limitations

Currently, there is no consensus on POTS subtyping, including the definition of the hyperadrenergic subtype. Therefore, it is not possible to calculate the sensitivity and specificity for different DBP_VM2l_rise_ thresholds. Conventional methods, such as ROC analysis, are thus not applicable for determining the optimal cut point. Our golden section search identification of an optimal cut point between patients with hyperadrenergic and non-hyperadrenergic POTS was based on an exploratory post hoc analysis, which should be confirmed by subsequent studies. The low sample size in the hyperadrenergic group limits the robustness of the proposed DBP_VM2l_rise_ cut point which needs to be confirmed by further studies. However, the 95% confidence interval for the group difference in psNVT (1.155–2.867 mmHg/spike·s^−1^) was narrow, entirely above zero, and even the lower bound represented a sizeable effect relative to the mean psNVT of the non-hyperadrenergic group. These indicated that our study had adequate power to detect meaningful differences in psNVT. We anticipate that our work will encourage further studies to standardize POTS subtyping. Also, the neurovascular transduction or psNVT in our study was estimated by the average response between MSNA and DBP only during the Valsalva. Additional research applying control theory and transfer function analysis between sympathetic activity and blood pressure is needed.

## Conclusion

We propose a clinical marker from the Valsalva maneuver, namely DBP_VM2l_rise_, as a surrogate for sympathetic function that is applicable for POTS subtyping. The relationship of DBP_VM2l_rise_ to sympathetic function has been confirmed by a strong correlation with B2B MSNA spike rate_VM2_ in healthy subjects. We found, by the golden section search method, that the DBP_VM2l_rise_ ≥ 15 mmHg is an optimal cut point to subtype hyperadrenergic POTS. The identified patients with hyperadrenergic POTS showed high sympathetic features in symptoms and other Valsalva metrics such as high Valsalva baseline SBP and short PRT, but similar MSNA activation to the non-hyperadrenergic group. The hyperadrenergic responses in these POTS are possibly due to improved neurovascular coupling, as suggested by higher psNVT compared to POTS with low DBP_VM2l_rise_. The current study introduced a practical clinical marker for POTS subtyping from Valsalva maneuver and suggested a novel pathophysiology of augmented neurovascular transduction for peripheral hyperadrenergic POTS, which would allow personalized treatment for these patients. The mechanisms of hyperadrenergic POTS and enhanced neurovascular coupling need to be elaborated in further studies.

## Supplementary Information

Below is the link to the electronic supplementary material.

Supplementary file1 (DOCX 503 KB)

Supplementary file2 (DOCX 27752 KB)

## Data Availability

Data are available upon request.
