# Supplementary material for: Hyperadrenergic postural tachycardia syndrome associated with augmented neurovascular transduction
Source: Clin Auton Res. 2026 Jan 14;36(2):271–83. doi: 10.1007/s10286-025-01183-z (PMC12931957; doi:10.1007/s10286-025-01183-z)
Supplement: Supplementary file 1 — Supplementary file1 (DOCX 503 KB) [file 10286_2025_1183_MOESM1_ESM.docx]

**Appendix 1**

**Method: DBP rise (DBP_VM2l_rise_) Cut Point by the Golden Section Search Optimization**

Our objective was to find the optimal cut point value for DBP_VM2l_rise_ to distinguish hyperadrenergic from non-hyperadrenergic POTS. We defined the optimal DBP_VM2l_rise_ cut point as the cut point where the highest correlations between MSNA and DBP_VM2l_rise_ were found in both hyperadrenergic (above the cut point value) and non-hyperadrenergic POTS (below the cut point value).

The interval between 0 and the maximum DBP_VM2l_rise_ of all POTS subjects was repeatedly partitioned by the ratio of r ($r=(\sqrt{5}-1)/2$) and $1-r$, resulting in two new evaluating points inside the interval. Then, the interval was narrowed down by moving one edge to the point that gave higher value of an objective function (Figure A1_1), which was the negative value of the product between the phase 2, excluding the last 5 s, beat-to-beat mean spike rate percentage difference from baseline (MSNA spike rate_VM2_) and DBP_VM2l_rise_ correlation coefficients of the two groups. The process was iterated until the interval width was less than a tolerance of 0.1 mmHg. The optimization was performed in a custom MATLAB script.


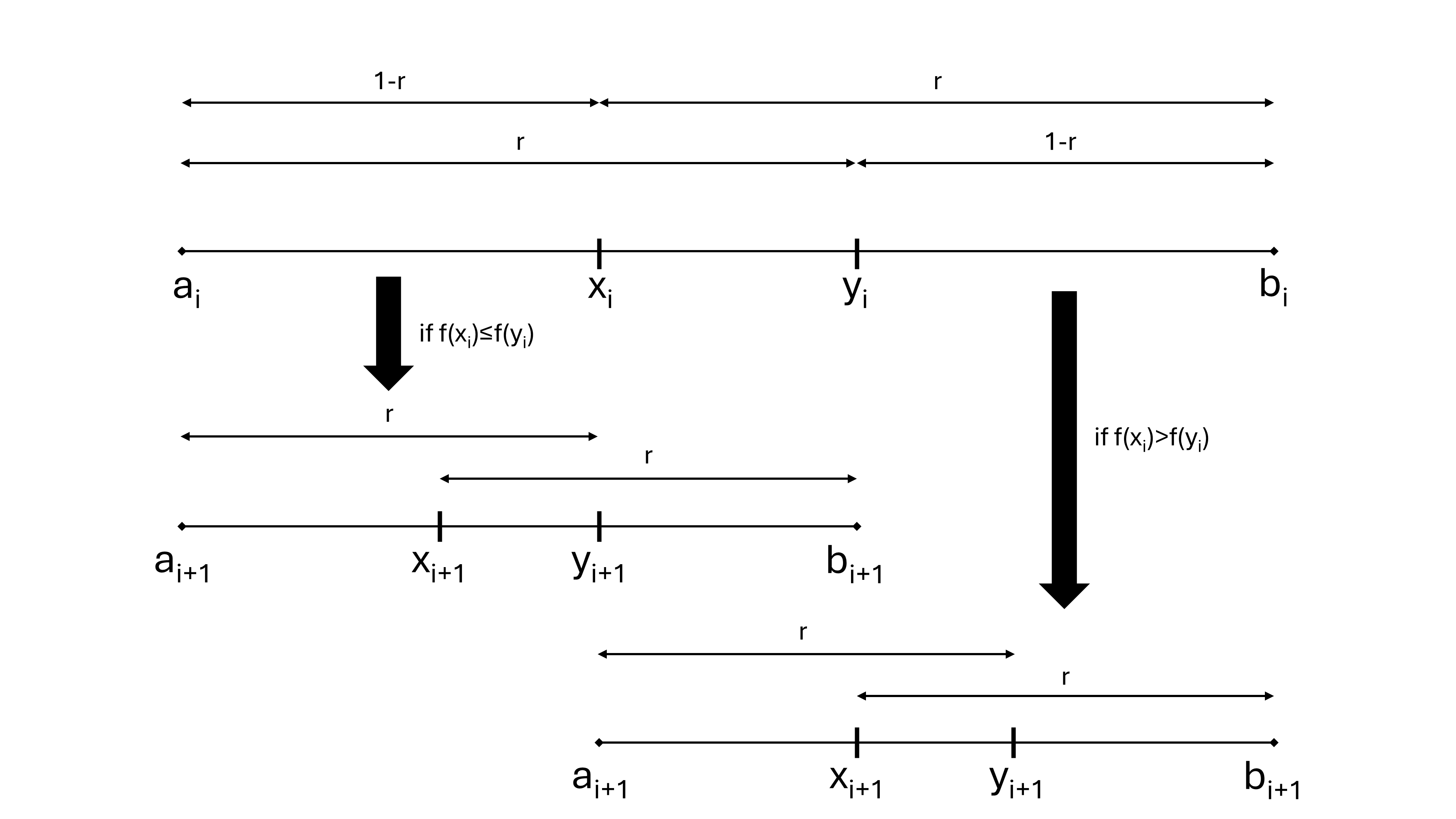


**Figure A1_1** Golden section search algorithm. a and b, search interval edges; x and y, newly added objective function evaluating points; r, golden section ratio = (√5-1)/2; f, objective function; i, number of iterations.

**MATLAB script**

%% golden section search

y=[1.615514572 21.62811674 9.375434722 6.143864598 20.72031559 27.18210778 ...

13.54206205 4.374645584 10.49991874 14.8788125 9.10843255 5.721576843 ...

13.81341719 8.556550952 41.75754324 6.460200592 8.713268277 16.05142575 ...

16.99338545]'; %vm2l_rise_dbp

x=[134.8813162 302.3001948 139.9970882 91.30475814 179.5869781 240.0750268 ...

142.0825756 116.696079 193.5072324 317.0260086 158.1487501 229.6402759 ...

260.81745 173.1470901 442.1507205 129.9418746 190.8754423 50.33413197 ...

75.36294889]'; %vm2fullminus5_mean_b2b_spkcountfreq_deltapercbsl

goldensectionsearch('objective_function',x,y,[0,max(y)],0.1) %choose interval between [0,max(y)] because vm2l_rise_dbp minimum is 0 (cannot be negative).

function total_corr = objective_function(cut,x,y)

% function maximizing product of two correlation coefficients

% Split data based on the cut point

% x is independent variable

% y is dependent variable

group1 = y < cut;

group2 = y >= cut;

% Ensure both groups have at least 2 samples

if sum(group1) < 3 || sum(group2) < 3

total_corr = Inf; % Penalize invalid splits

return;

end

% Compute correlation in both groups

corr1 = corr(x(group1), y(group1));

corr2 = corr(x(group2), y(group2));

% Objective: maximize average correlation

total_corr = -abs(corr1 * corr2); %put negative to make it a maximize problem

end

function goldensectionsearch(func,x,y,ab,tolx)

%input

%func: non-linear function to be minimized

%ab: bracketing interval [a,b]

%tolx: tolerance for estimating the minimum

r=(sqrt(5)-1)/2; %interval reduction for golden ratio;

k=0; %counter

%Bracketing interval

a=ab(1);

b=ab(2);

fa=feval(func,a,x,y); %f(x) at x=a

fb=feval(func,b,x,y); %f(x) at x=b

%interior point

x1=(1-r)*(b-a)+a;

x2=r*(b-a)+a;

fx1=feval(func,x1,x,y);

fx2=feval(func,x2,x,y);

%iterative solution

while b-a>tolx

fprintf('iteration %2d: a=%.4f b=%.4f f(a)=%.4f f(b)=%.4f \n',[k,a,b,fa,fb])

if fx1>fx2

a=x1; %move left border to the right

fa=fx1;

x1=x2;

fx1=fx2;

x2=r*(b-a)+a;

fx2=feval(func,x2,x,y);

else

b=x2; %move right border to left

fb=fx2;

x2=x1;

fx2=fx1;

x1=(1-r)*(b-a)+a;

fx1=feval(func,x1,x,y);

end

k=k+1;

end

fprintf('iteration %2d: a=%.4f b=%.4f f(a)=%.4f f(b)=%.4f \n',[k,a,b,fa,fb])

end

**Results**

The search converged to an interval width of 0.08 mmHg (less than the set threshold of 0.1 mmHg) within 13 iterations. The final cut point found was 14.9314 mmHg which gave the correlation coefficient between MSNA spike rate_VM2_ and DBP_VM2l_rise_ for the group above the cut point at r=0.902 (p=0.014) and for the group below the cut point at r=0.629 (p=0.021) resulting in the highest product of the two coefficients at 0.5674.

Printed results from the search

>> dbp_goldenratio

iteration 0: a=0.0000 b=41.7575 f(a)=Inf f(b)=Inf

iteration 1: a=0.0000 b=25.8076 f(a)=Inf f(b)=Inf

iteration 2: a=9.8576 b=25.8076 f(a)=-0.1729 f(b)=Inf

iteration 3: a=9.8576 b=19.7152 f(a)=-0.1729 f(b)=-0.0645

iteration 4: a=13.6229 b=19.7152 f(a)=-0.1455 f(b)=-0.0645

iteration 5: a=13.6229 b=17.3882 f(a)=-0.1455 f(b)=-0.0645

iteration 6: a=13.6229 b=15.9500 f(a)=-0.1455 f(b)=-0.5674

iteration 7: a=14.5118 b=15.9500 f(a)=-0.3312 f(b)=-0.5674

iteration 8: a=14.5118 b=15.4006 f(a)=-0.3312 f(b)=-0.5674

iteration 9: a=14.8513 b=15.4006 f(a)=-0.3312 f(b)=-0.5674

iteration 10: a=14.8513 b=15.1908 f(a)=-0.3312 f(b)=-0.5674

iteration 11: a=14.8513 b=15.0611 f(a)=-0.3312 f(b)=-0.5674

iteration 12: a=14.8513 b=14.9810 f(a)=-0.3312 f(b)=-0.5674

iteration 13: a=14.8513 b=14.9314 f(a)=-0.3312 f(b)=-0.5674
