# Supplementary material for: Hyperadrenergic postural tachycardia syndrome associated with augmented neurovascular transduction
Source: Clin Auton Res. 2026 Jan 14;36(2):271–83. doi: 10.1007/s10286-025-01183-z (PMC12931957; doi:10.1007/s10286-025-01183-z)
Supplement: Supplementary file 2 — Supplementary file2 (DOCX 27752 KB) [file 10286_2025_1183_MOESM2_ESM.docx]

**Appendix 2: Effects of Moxonidine on POTS During Valsalva Maneuver**

This appendix explains an additional study of the randomized treatment trial using moxonidine in postural tachycardia syndrome (POTS) patients. Moxonidine is a specific imidazoline I1 receptor and α2 adrenergic receptor agonist that inhibits sympathetic outflow from the rostral ventrolateral medulla (RVLM)^[1,2]^. It is used as a centrally acting antihypertensive drug that can reduce blood pressure, plasma norepinephrine, and MSNA. We would like to evaluate the effect of central sympatholysis with moxonidine on Valsalva sympathetic markers in POTS patients after they were subtyped by our optimized diastolic blood pressure rise during the late phase 2 of Valsalva (DBP_VM2l_rise_) cut point.

**Methods**

After the initial baseline study, POTS patients underwent a double-blind, randomized, placebo-controlled, 2-day crossover trial with 0.2-0.4 mg PO moxonidine. Medications were prepared by the Vanderbilt Investigational Pharmacy. After 2 hours of drug administration, the Valsalva maneuver was performed with measurements of continuous and intermittent blood pressure (BP), electrocardiogram (ECG), and muscle sympathetic nerve activity (MSNA). Patients repeated the study on the following day with the other drug, moxonidine or placebo (Figure A2_1).


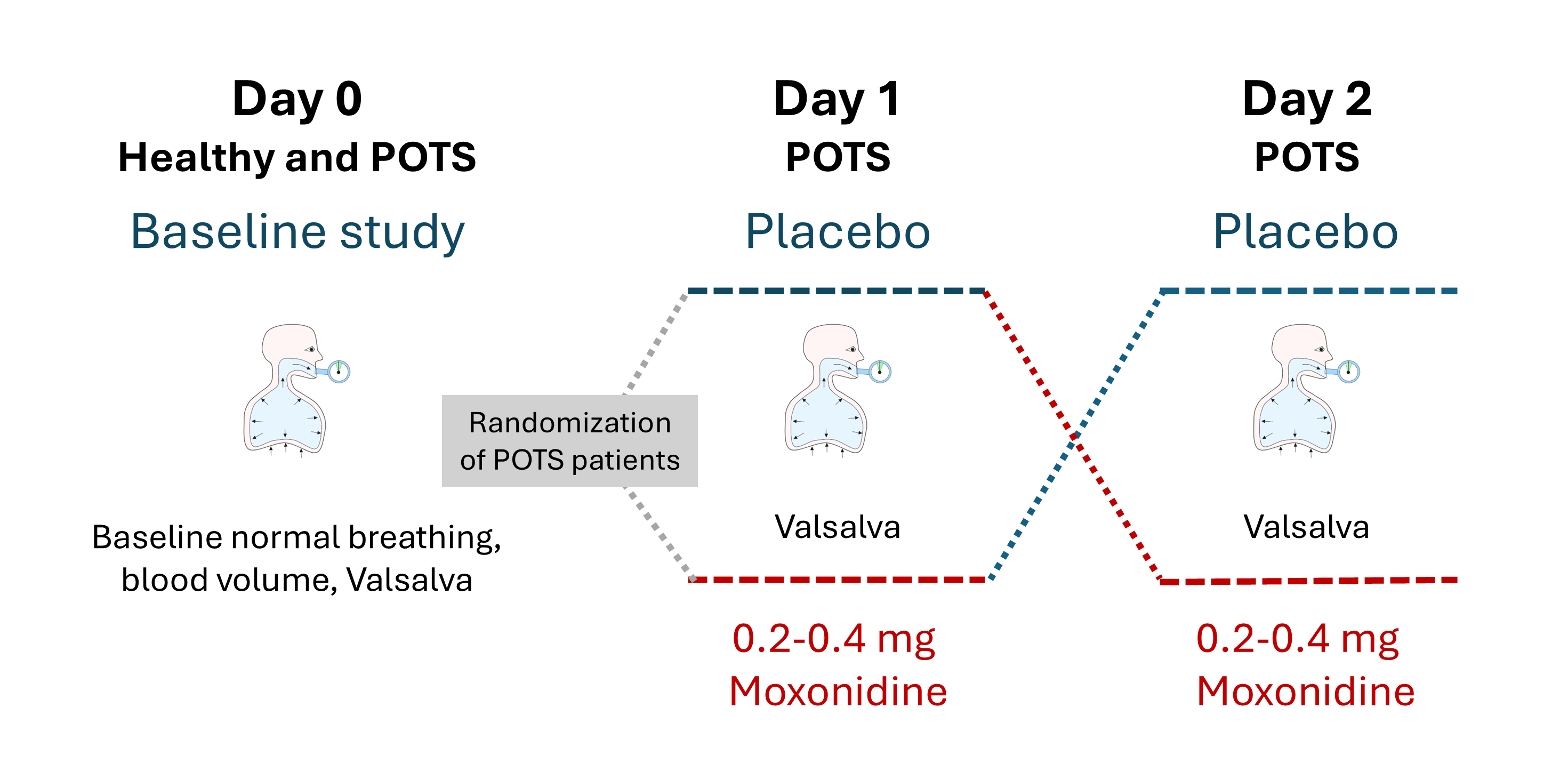


**Figure A2_1** Protocol. All participants performed baseline normal breathing, carbon monoxide rebreathing blood volume measurement, and Valsalva maneuver on day 0. POTS subjects further performed this maneuver on the 2-day randomized placebo-moxonidine trial on study day 1 and study day 2.

d

POTS patients were grouped into hyperadrenergic POTS (DBP_VM2l_rise_ ≥ 15 mmHg) and non-hyperadrenergic POTS (DBP_VM2l_rise_ < 15 mmHg) based on their data during the baseline study (day 0). Statistical analyses between the two groups were performed using the unpaired t-test for normally distributed data and the Wilcoxon rank-sum test for non-normally distributed data. Paired comparisons between placebo and moxonidine effects were analyzed using the paired t-test and the Wilcoxon signed rank test for data with normal and non-normal distribution, respectively.

**Results**

Fourteen patients with POTS completed the trial with moxonidine, but ten of them had successful microneurography recordings in both visits (Table A2_1). Compared to placebo, moxonidine reduced Valsalva baseline SBP (110±14 vs. 104±16 mmHg, p=0.042) and DBP (71±10 vs. 67±10 mmHg, p=0.036). However, BP, HR, and MSNA changes during the Valsalva maneuver did not generally differ between the treatment groups (Table A2_1).

Four patients with POTS, who had a DBP_VM2l_rise_ ≥ 15 mmHg in the baseline study, completed the moxonidine trial. Compared to placebo, moxonidine in these patients tended to decrease the Valsalva baseline SBP (119±20 vs. 111±21 mmHg, p=0.250), DBP_VM2l_rise_ (27±12 vs. 18±20 mmHg, p=0.175) and the SBP overshoot (35±28 vs. 28±30 mmHg, p=0.683), and tended to increase the PRT (3.87±6.57 vs. 9.24±15.90 seconds, p=0.250). In general, BP and MSNA (but not HR) during Valsalva tended to be lower in the moxonidine group, but none of the pairwise comparisons were statistically significant (Figure A2_2).

The magnitude of differences when having moxonidine versus placebo (moxonidine minus placebo) also tended to be larger in the DBP_VM2l_rise_ ≥ 15 group (n=4) than the DBP_VM2l_rise_ < 15 mmHg group (n=10) for most Valsalva parameters including Valsalva baseline SBP (-8±12 vs. -5±10 mmHg, p=0.688), early phase 2 duration (Duration_VM2e_, 3.02±2.58 vs. -0.52±1.29 s, p=0.004), and pressure recovery time (PRT, 5.37±9.34 vs. 0.40±0.36 s, p=0.188). The DBP_VM2l_rise_ suppressed by moxonidine seemed to be more pronounced in hyperadrenergic POTS than in the other group as shown in both absolute value changes (-9±11 vs 1±8 mmHg, p=0.060) and area under the curve changes (-37.46±55.04 vs 2.71±28.26 mmHg∙s, p=0.090, Figure A2_3).

**Figure A2_2** Overlay plots of heart rate (HR, top, n=4), finger blood pressure (FBP, middle, n=4) where upper line represents systolic blood pressure and lower line represent diastolic blood pressure, and MSNA spike rate (bottom, n=3) changes during Valsalva maneuver in possible hyperadrenergic POTS having placebo (blue) and moxonidine (red). Light lines represent individual responses. Dark lines represent average responses. The Valsalva straining begins at time 0 s and last for 15 s (gray area).


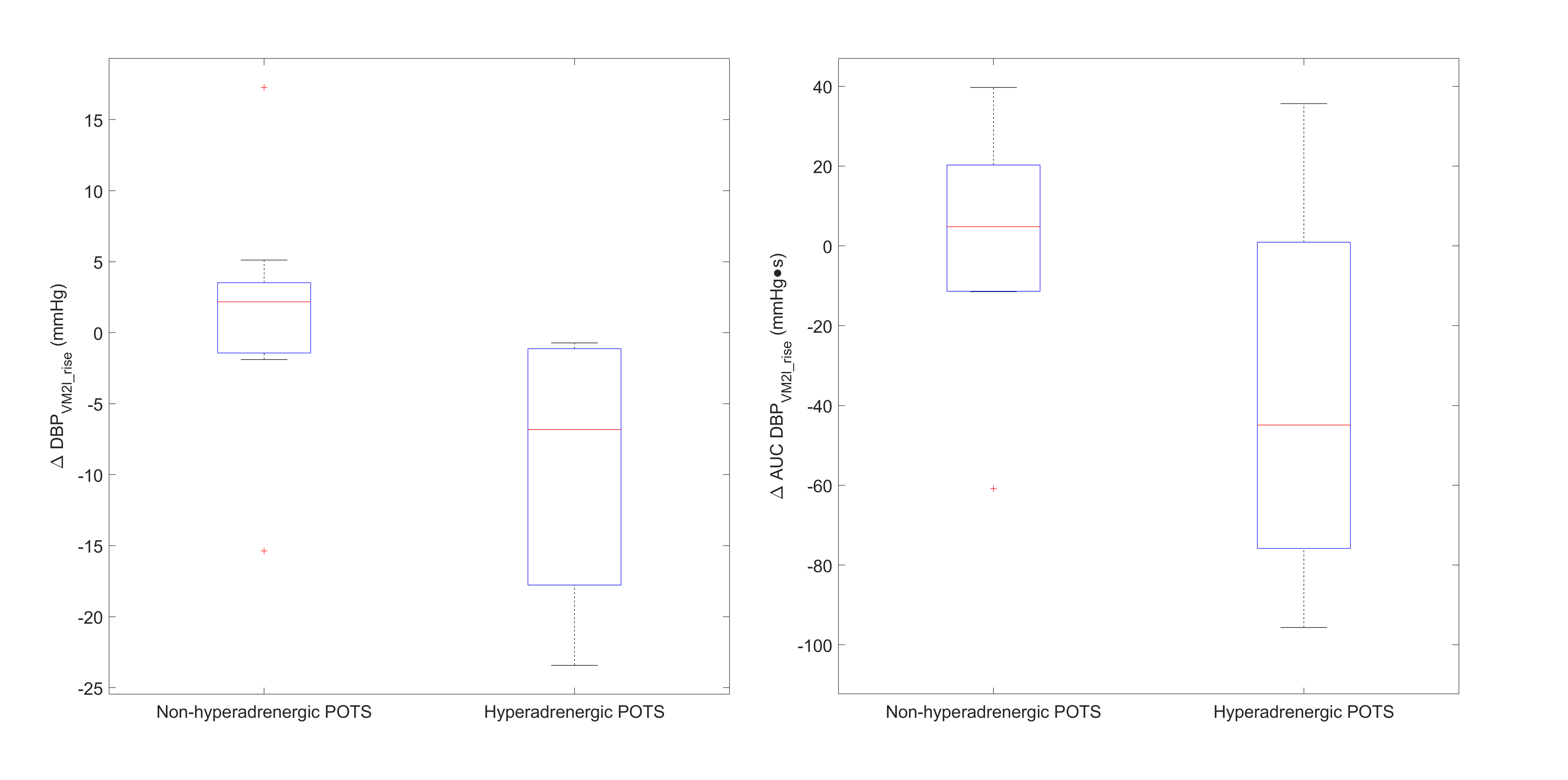


**Figure A2_3** Effects of moxonidine on late phase 2 DBP rise (DBP_VM2l_rise_) in non-hyperadrenergic POTS (n=10) and hyperadrenergic POTS (n=4) when compared to placebo (moxonidine minus placebo). Left: comparison of DBP_VM2l_rise_ differences (∆DBP_VM2l_rise_, p=0.060). Right: comparison of area under the curve of DBP_VM2l_rise_ differences (∆AUC DBP_VM2l_rise_, p=0.090). The box plots represent data between 25^th^ to 75^th^ percentile while the red horizontal line indicates the median. The whiskers extend to the most extreme data points excluding outliers. Outliers are plotted individually using the '+' marker symbol.

**Table A2_1** Valsalva maneuver responses comparison between POTS having moxonidine versus placebo.

|  | Placebo (n=14)  MSNA related values, n=10 | Moxonidine (n=14)  MSNA related values, n=10 | p-value |
| --- | --- | --- | --- |
| Valsalva Baseline |  |  |  |
| SBP (mmHg) | 110±14 | 104±16 | 0.042 |
| DBP (mmHg) | 71±10 | 67±10 | 0.036 |
| HR (beat/min) | 75±12 | 78±16 | 0.078 |
| MSNA spike rate (spike/s) | 13.57±6.49 | 13.77±6.72 | 0.849 |
| Phase 2 |  |  |  |
| SBP_endVM2e_ (mmHg) | 79±10 | 74±10 | 0.088 |
| DBP_endVM2e_ (mmHg) | 64±10 | 58±7 | 0.058 |
| HR_endVM2e_ (beat/min) | 98±16 | 100±21 | 0.450 |
| MSNA spike rate_meanVM2e_ (spike/s) | 26.45±13.99 | 27.14±12.08 | 1.000 |
| SBP_VM2e_deltabsl_ (mmHg) | -31±8 | -30±10 | 0.684 |
| DBP_VM2e_deltabsl_ (mmHg) | -8±6 | -10±6 | 0.274 |
| Duration_VM2e_ (s) | 7.65±2.22 | 8.14±2.79 | 0.715 |
| SBP slope_VM2e_ (mmHg/s) | -4.46±1.98 | -4.35±2.53 | 0.812 |
| Cardiovagal BRS_VM2e_ (ms/mmHg) | 5.53±3.56 | 5.52±4.26 | 0.808 |
| SBP_endVM2l_ (mmHg) | 98±26 | 87±22 | 0.056 |
| DBP_endVM2l_ (mmHg) | 76±16 | 70±15 | 0.114 |
| HR_endVM2l_ (beat/min) | 108±19 | 113±24 | 0.305 |
| MSNA spike rate_meanVM2l_ (spike/s) | 37.55±15.88 | 34.72±15.78 | 0.778 |
| SBP_VM2l_rise_ (mmHg) | 19±19 | 15±16 | 0.358 |
| DBP_VM2l_rise_ (mmHg) | 15±12 | 14±13 | 0.855 |
| DBP slope_VM2l_ (mmHg/s) | 3.02±2.17 | 2.27±1.55 | 0.252 |
| csBRS (spike∙s^-1^/mmHg) | 1.34±1.41 | 2.21±2.32 | 0.922 |
| psNVT (mmHg/spike∙s^-1^) | 1.30±0.94 | 1.76±1.83 | 0.475 |
| Phase 3 |  |  |  |
| SBP_endVM3_ (mmHg) | 84±22 | 75±16 | 0.047 |
| DBP_endVM3_ (mmHg) | 67±16 | 61±11 | 0.060 |
| HR_endVM3_ (beat/min) | 110±18 | 114±22 | 0.343 |
| Phase 4 |  |  |  |
| SBP_maxVM4_ (mmHg) | 138±40 | 126±31 | 0.112 |
| DBP_maxVM4_ (mmHg) | 88±19 | 81±17 | 0.094 |
| VM ratio | 2.11±0.39 | 2.06±0.46 | 0.567 |
| PRT (s) | 2.78±3.34 | 4.60±8.28 | 0.001 |
| SBP overshoot (mmHg) | 28±28 | 22±18 | 0.303 |
| Cardiovagal BRS_VM4_ (ms/mmHg) | 12.16±4.38 | 11.29±3.97 | 0.416 |

Values presented as mean ± SD; SBP, systolic blood pressure; DBP, diastolic blood pressure; HR, heart rate; MSNA, muscle sympathetic nerve activity; BRS, baroreflex sensitivity; csBRS, central sympathetic baroreflex sensitivity; psNVT, peripheral sympathetic neurovascular transduction; VM ratio, Valsalva ratio; PRT, pressure recovery time; deltabsl, difference from baseline; VM2e, VM2l, VM3, VM4 stand for Valsalva early phase 2, late phase 2, phase 3, and phase 4, respectively.

**Discussion**

In the whole group of patients with POTS, moxonidine decreased Valsalva baseline BP, but the Valsalva responses were not different from placebo, except for a longer PRT (Table A2_1). A previous study showed that moxonidine can reduce BP in hypertensive patients despite insignificant MSNA burst rate reduction, similar to our results^[2]^. Negative results between moxonidine and placebo may be due to heterogeneity in POTS subtypes.

We performed a subgroup analysis only on hyperadrenergic POTS, identified by our proposed DBP_VM2l_rise_ ≥ 15 mmHg, which could be a target group for sympatholytic treatment. Moxonidine seems to effectively suppress sympathetic activity in hyperadrenergic POTS, as shown by reduced Valsalva baseline SBP, decreased SBP overshoot, prolonged PRT, and most importantly, lower DBP_VM2l_rise_. The magnitude of changes in these sympathetic markers also tended to be larger in the hyperadrenergic group. Moxonidine reduced the DBP_VM2l_rise_ and the area under the curve of the DBP_VM2l_rise_ only in the hyperadrenergic group (Figure A2_3), but this did not reach statistical significance due to the small sample size of hyperadrenergic POTS. Our preliminary results confirm the hyperadrenergic state of POTS with high DBP_VM2l_rise_ and suggest that moxonidine could selectively benefit hyperadrenergic POTS.

**Limitations**

Our interpretations were limited by the small sample size, which made statistical tests infeasible for pairwise comparisons within hyperadrenergic POTS. The validity of DBP_VM2l_rise_ as a clinical biomarker of hyperadrenergic POTS could be further validated by correlating it with a positive response to central sympatholytics in a larger randomized controlled trial.

**References**

1. Morris, S., & Reid, J. (1997). Moxonidine: A review. *Journal of Human Hypertension*, *11*(10), 629–635. https://doi.org/10.1038/sj.jhh.1000518

2. Wenzel, R. R., Spieker, L., Qui, S., Shaw, S., Lüscher, T. F., & Noll, G. (1998). I_1_ -Imidazoline Agonist Moxonidine Decreases Sympathetic Nerve Activity and Blood Pressure in Hypertensives. *Hypertension*, *32*(6), 1022–1027. https://doi.org/10.1161/01.HYP.32.6.1022
